# Retrospective analysis of safety and efficacy of enhanced recovery pathways in stage II–III colorectal cancer patients submitted to surgery and adjuvant therapy

**DOI:** 10.1186/s12957-021-02203-8

**Published:** 2021-04-06

**Authors:** Baoxin Wang, Zhenming Wu, Rui Zhang, Yue Chen, Jiuxing Dong, Xiuheng Qi

**Affiliations:** Department of Oncology, Hebei Petrochina Central Hospital, Heibei, Langfang, 065000 China

**Keywords:** Colorectal cancer, Chemotherapy, Fast-track, Hospital stay, Laparoscopy, Open surgical procedure

## Abstract

**Background:**

The American Society of Colon and Rectal Surgeons is suggesting laparoscopic surgeries for colorectal cancer. Conventional perioperative procedures like long preoperative fasting and bowel procedures are not useful and harmful to patients undergoing surgeries for colorectal cancer. The objectives of the study were to compare surgery outcomes, hospital stays, and survival of patients who received fast-track (laparoscopy/open) surgical procedure followed by chemotherapy against those who received conventional (laparoscopy/open) surgical procedure followed by chemotherapy for colorectal cancer.

**Methods:**

The study analyzes the outcomes of a total of 542 colorectal cancer (preoperative biopsies stage II or III) patients submitted to surgery and adjuvant chemotherapy. The study cohort is retrospectively subdivided in 4 groups submitted to open or laparoscopic resection with or without fast-track protocol appliance and two different chemotherapy regimens. Patients who ended up being TNM stage I have not received the adjuvant chemotherapy.

**Results:**

The fast-track surgical procedure had shorter total hospital stays and postoperative hospital stays than the conventional surgical procedures. Flatus resumption time, the time until first defecation, and intraoperative blood loss were shorter for the fast-track surgical procedures than the conventional surgical procedures. Those surgery outcomes were also shorter for the fast-track laparoscopy than the open fast-track. Resumption of a fluid diet and ambulation onset time were shorter for the fast-track surgical procedures than the conventional surgical procedures. The surgical checkpoints that were compliance by patient of fast-track surgeries were significantly fewer than those of the conventional surgeries. Clinically significant difference for QLQ-C30/CR38 score after chemotherapy was reported between patients who received open conventional surgeries and those patients who received fast-track laparoscopy (59.63 ± 2.26 score/patient vs. 71.67 ± 5.19 score/patient). There were no significant differences for the number of patients with any grade adverse effects (*p* = 0.431) or with grade 3–4 adverse effects (*p* = 0.858), and the disease-free and overall survival among cohorts.

**Conclusions:**

The fast-track surgical procedure is effective and safe even in a multidisciplinary scenario as colorectal cancer treatment in which surgery is only a part of management.

**Level of evidence: III:**

Technical efficacy stage: 4.

## Background

Colorectal cancer was the third most common cancer worldwide in 2017 [1] and the second most common cancer worldwide in 2020 [2]. Colorectal cancer is the fifth most common cancer responsible for death [3]. Patients with colorectal cancer are generally cured by radical surgery followed by chemotherapy and/or radiotherapy regimens [4]. The American Society of Colon and Rectal Surgeons is suggesting laparoscopic surgeries for colorectal cancer [5]. Conventional perioperative procedures like long preoperative fasting and bowel procedures are not useful and even harmful to patients undergoing surgeries for colorectal cancer [6, 7]. Fast-track surgery includes several perioperative interventions for the enhancement of recovery of patients [4].

LAFA-study [8] and EnROL trial [9] on the Caucasian population and the randomized trials on the Chinese population [4, 10–13] are reporting that the fast-track surgery is effective for colorectal cancer patients regarding postoperative recoveries. However, trials on the Chinese population [4, 10–13] are not comprehensive, some are with a small number of patients and did not report preoperative procedural details, and only 1-week postoperative period are studied. However, patients may require 6 months of chemotherapy after surgery [4]. Also, some of the surgical procedures recommended by LAFA study [8] and EnROL trial [9] are difficult to implement in the Chinese population.

Therefore, the fast-track surgical model for multidisciplinary treatment protocol [14] of the Chinese population is taken into consideration for surgeries for colorectal cancer in several institutes of China, which includes more conservative surgeries than those of the Western countries. Also, it covers the overall treatment process.

The primary aim of the retrospective study was to compare surgical checkpoints that were compliance by patients, surgery outcomes, and length of hospital stays of patients who underwent fast-track surgery (laparoscopy/open surgical procedure) against those who underwent conventional surgery (laparoscopy/open surgical procedure) for colorectal cancer. The secondary aim of the study was to compare chemotherapy-related adverse events, quality of life after chemotherapy regimens, and survival during follow-up of patients who received capecitabine and oxaliplatin chemotherapy against those who received leucovorin, fluorouracil, and oxaliplatin chemotherapy regimens after surgery for colorectal cancer.

## Methods

### Study population

Patients (> 18 years old) with pathologically confirmed colon or upper rectal (the distance of tumor lower margin from anus > 12 cm) cancer (preoperative biopsies stage II or stage III) from 15 January 2018 to 25 December 2019 were included in the analysis. Patients whose tumor were removed by the endoscopic mucosal procedure(s), patients who are pregnant, patients who have a spinal deformity, and patients who had undergone surgery followed by chemotherapy of mid-low rectal cancer were excluded from the analysis, as this would have greatly affected the main outcomes of the study.

### Sample size calculation

Based on patients need for adjuvant chemotherapy, power of 80% (*β* = 0.2), 5% of two-sided type-I error (*α* = 0.05), and 95% of confidence level [4], there was a need of a minimum of 500 patients with at least 125 patients in each cohort.

### Cohort

A total of 125 patients were subjected to fast-track laparoscopic surgery followed by 8 cycles (21 days in between two cycles) of 850 mg–1 g/m^2^ capecitabine (Xeloda, F. Hoffmann-La Roche AG, Grenzacherstrasse, Basel, Switzerland) and 130 mg/m^2^ oxaliplatin (Eloxatin, Sanofi-aventis, rue La Boetie, Paris, France) chemotherapy (if required) (FTLCO cohort). A total of 137 patients were subjected to open fast-track surgery followed by 8 cycles (21 days in between two cycles) of 850 mg–1 g/m^2^ capecitabine and 130 mg/m^2^ oxaliplatin chemotherapy (if required) (OTLCO cohort). A total of 142 patients were subjected to conventional laparoscopic surgery followed by 12 cycles (15 days in between two cycles) of 400 mg/m^2^ leucovorin (Wellcovorin, Wyeth Pharmaceuticals Limited, Overland Park, Kansas, USA), 400 mg/m^2^ 5-fluorouracil (Adrucil®, Teva Pharamceuticals Inc., North Wales, PA, USA), and 85 mg/m^2^ oxaliplatin chemotherapy (if required) (CSLFO cohort). A total of 138 patients subjected to open conventional surgery followed by 12 cycles (15 days in between two cycles) of 400 mg/m^2^ leucovorin, 400 mg/m^2^ 5-fluorouracil, and 85 mg/m^2^ oxaliplatin chemotherapy (if required) (OSLFO cohort). The differences between fast-track and the conventional surgical procedures are summarized in Table 1 [14]. All surgical procedures were performed according to the National Comprehensive Cancer Network Clinical Practice Guidelines in Oncology® Colon Cancer V.2.2018 [15] by colorectal surgeons (with a minimum of 3 years of institutional training of abdominal surgeries) of the institutes. The chemotherapy was given by nursing staff (minimum 3 years of experiences; aware of the type of surgeries) of the institutes. The different adjuvant therapies to the factorial design of the treatment subgroups were added because of the instruction of the institute (institutional protocol).

**Table 1:** The differences between fast-track and the conventional surgical procedures for colorectal cancer

Parameters	Fast-track surgical procedure	The conventional surgical procedure
Preadmission	Mental optimism.	No mental optimism.
Counseling	Pre-assessment for risk adjustment.	Pre-assessment for risk adjustment.
	Information for thoracic epidural and general combined anesthesia.	No information for combined anesthesia.
	Information for fast-track surgical procedure and consent.	Information for the conventional surgical procedure and consent.
Surgical preparation	Bowel preparation.	Bowel preparation.
	Enemas.	Enemas.
	The last meal: 2 h before surgery.	The last meal: 10 h before surgery.
	Complete enteral nutritional. 500 mL 10% glucose 2–3 h before surgery (if required).	No oral intake on the day of surgery.
Gastrointestinal decompression	~30 min before surgery by the nasogastric tube.	~30 min before surgery by the nasogastric tube.
Perioperative management	Thoracic epidural anesthesia.	No thoracic epidural anesthesia.
	Balanced combination with the general anesthesia.	Normal general anesthesia.
	Mechanical ventilation.	Mechanical ventilation.
General anesthesia	Propofol and rocuronium	Propofol and rocuronium
Opioid	Morphine injection as low as possible.	No restriction of morphine injection use.
Monitoring	Hemodynamic parameters.	Hemodynamic parameters.
Prophylaxis	Intravenous antibiotic(s).	Intravenous antibiotic(s).
Surgery	Laparoscopy/open surgical procedure.	Laparoscopy/open surgical procedure.
Warming	Yes.	No.
Drains	Minimal use.	Regular use.
Fluid infusion	≤ 1.5 L.	No restriction.
Pain management	Epidural analgesia + paracetamol infusion.	Sufentanil.
Postoperative diet	1-piece chewing gum three times in a day (if required).	Chewing gum.
	200 mL 10% glucose within 1 day after operation (if required).	Fasting until flatus.
	Liquid diet on the next day of operation (if required).	Liquid diet after flatus.
	The rehabilitation of diet as early as possible.	Diet after defecation.
Intravenous fluid infusion	Maximum for 3 days or until nutritional emulsion administered	High energy fluid on daily basis (infusion) until oral intake.
Energy	25–30 kcal/kg/day.	25–30 kcal/kg/day.
Nasogastric tube	Removed after surgery.	Removed after the first flatus.
Urethral catheter	Removed within 2 days after surgery.	Removed when automatic micturition feeling.
Ambulation	Within 24 h after surgery, ≥ 1 h/day and gradually increased.	No ambulation schemes.
Adjuvant chemotherapy (if required; institutional protocol)	8 cycles of capecitabine and oxaliplatin; every 21 days	12 cycles of leucovorin, fluorouracil, and oxaliplatin; every 15 days
Hospitalization for chemotherapy	1 day	3 days

All surgical procedures were performed according to the National Comprehensive Cancer Network Clinical Practice Guidelines in Oncology® Colon Cancer V.2.2018.

Data regarding surgical checkpoints, hospital stays, outcomes for surgery, chemotherapy-related adverse events, quality of life after chemotherapy, and survival during follow-up of patients were retrospectively collected from medical records of the institute and analyzed.

### Surgical checkpoints

The number of checkpoints to be compliance by the planned procedures was noted. There was a maximum of 13 checkpoints. If the patient was violated 10 or more checkpoints, then it was considered as the patient was not received planned allocated intervention(s). The checkpoints were (1) preadmission and counseling, (2) surgical preparation, (3) gastrointestinal decompression, (4) perioperative management (general and epidural anesthesia), (5) opioid and prophylaxis administration, (6) hemodynamic parameters monitoring, (7) warming, (8) grains, (9) fluid infusion, (10) Pain management, (11) postoperative diet, (12) removal of nasogastric tube and urethral catheter, and (13) ambulation.

### Hospital stays

Time from hospitalization to discharge was considered as length of hospital stay. Time after the operation to discharge of hospital was considered as postoperative hospital stays. Chemotherapy-related hospital stays were not included in the appropriate groups.

### Outcomes for surgery

Intraoperative blood loss, the readmission within 30 days, surgical cost, self-reported flatus resumption time, the time until the first defecation, resumption of a fluid diet time (time to take nutrition and fluid balance seriously), and ambulation onset time (the time required for the first the act, action, or an instance of moving after surgery) were retrospectively collected from the patients’ records of the institute.

### Chemotherapy and chemotherapy-related adverse events

The adjuvant chemotherapy was administered in a later admission after a full recovery, usually 3–4 weeks after post-operative discharge. Patients who had the surgical pathological TNM stage (Tumor, Node, and Metastasis stage; related to AJCC staging system) II, III, or IV have received all cycles of chemotherapy. Patients who ended up being TNM stage I have not received the adjuvant chemotherapy. The National Cancer Institute Common Terminology Criteria for Adverse Events v4.03 was preferred for evaluation of the chemotherapy-related adverse events [16]. The data of the cost of chemotherapy was collected from pharmacy and hospital records of patients.

### Quality of life

The quality of life of patients who received treatment were assessed by the trained instructors (3 years of experiences) of institutes using European Organization for Research and Treatment questionnaires before treatment and after completion of all chemotherapy regimens. The median differences of QLQ-C30/CR38 (quality of life questionnaires-cancer specific 30/colorectal cancer specific 38) score of 10 or more points were considered as clinically significant differences [17].

### Statistical analysis

InStat 3.01 GraphPad, San Diego, USA, was used for statistical analyses purpose. Categorical and ordinal variables are presented as frequency (percentages) and continuous variables are presented as mean ± standard deviation (SD). Categorical and ordinal variables were analyzed by the chi-square test for independence or Fisher’s exact test and continuous variables were analyzed by one-way analysis of variance (ANOVA). The Tukey test (considering critical value (*q*) > 3.633 as significant) was used for post hoc analysis. All results were considered significant if *p* value was reported less than 0.05.

## Results

### Study population

**Fig. 1:**
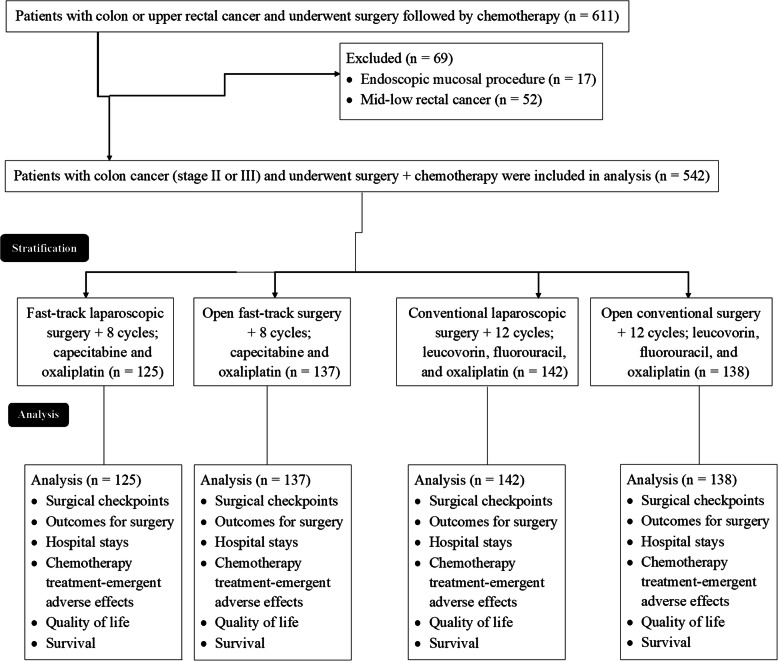
The treatment chart of colorectal cancer patients

From 15 January 2018 to 25 December 2019, a total of 611 patients (> 18 years old) were pathologically diagnosed as colon or upper rectal (the distance of tumor lower margin from anus > 12 cm) cancer and underwent surgery (fast-tract or conventional) followed by chemotherapy at the Hebei Petrochina Central Hospital, Langfang, Heibei, China, and the referring hospitals. Among 611 patients, the tumor of 17 patients was removed by the endoscopic mucosal procedure and 52 patients had faced surgeries followed by chemotherapy of mid-low rectal cancer. Therefore, data of these patients (*n* = 69) were excluded from analysis. Data of 542 patients with colon cancer (preoperative biopsies stage II or stage III) and underwent fast-tack or conventional surgeries (laparoscopy/open surgical procedure) followed by chemotherapy regimens (capecitabine and oxaliplatin or leucovorin, fluorouracil, and oxaliplatin) were included in the analysis. The treatment chart of the included patients is presented in Fig. 1.

### Demographic and clinical characteristics

All patients had the American Society of Anesthesiologists status I or II (except 2 patients) at the time of surgeries. There were no statistical differences for the demographic and clinical characteristics of patients at the time of surgeries (*p* > 0.05 for all, Table 2) among cohorts.

**Table 2:** The demographic and clinical characteristics of patients at the time of surgery

Parameters		Cohorts				Comparisons
		FTLCO	OTLCO	CSLFO	OSLFO	
Surgery		Fast-track laparoscopy	Open fast-track	Conventional laparoscopy	Open conventional	
Adjuvant chemotherapy (if required)		8 cycles of capecitabine and oxaliplatin; every 21 days	8 cycles of capecitabine and oxaliplatin; every 21 days	12 cycles of leucovorin, fluorouracil, and oxaliplatin; every 15 days	12 cycles of leucovorin, fluorouracil, and oxaliplatin; every 15 days	
Numbers of patients underwent surgeries		125	137	142	138	*p* value
Age (years)	Minimum	52	51	53	53	0.129
	Maximum	67	68	69	69	
	Mean ± SD	56.22±7.15	57.18±8.19	57.65±6.15	58.45±9.18	
Sex	Male	75 (60)	89 (65)	91 (64)	86 (62)	0.848
	Female	50 (40)	48 (35)	51 (36)	52 (38)	
Body mass index (kg/m^2^)	Minimum	21	21	21	21	0.904
	Maximum	26	26	26	26	
	Mean ± SD (kg/m^2^/patient)	24.12±2.13	24.21±2.01	24.32±2.45	24.27±2.45	
The American Society of Anesthesiologists status	I	99 (79)	121 (88)	125 (88)	122 (88)	0.219
	II	25 (20)	15 (11)	17 (12)	16 (12)	
	III	1 (1)	1 (1)	0 (0)	0 (0)	
Carcinoembryonic antigen (ng/mL/patient)		6.71±1.12	6.52±1.25	6.45±1.35	6.58±1.56	0.441
Carbohydrate antigen 19–9 (ng/mL)		35.12±7.11	36.21±6.15	37.15±8.11	36.15±8.15	0.177
Site	Rectum	38 (30)	41 (30)	41 (28)	40 (29)	0.992
	Sigmoid colon	35 (28)	40 (29)	38 (27)	41 (29)	
	Ascending colon	24 (19)	27 (20)	25 (18)	26 (19)	
	Descending colon	17 (14)	17 (12)	18 (13)	15 (11)	
	Transverse colon	11 (9)	12 (9)	20 (14)	16 (12)	
Maximum tumor diameter (cm/patient)		4.11±0.18	4.15±0.19	4.13±0.21	4.09±0.25	0.103
Pathologic type	Adenocarcinoma	107 (86)	115 (84)	121 (85)	122 (88)	0.753
	Mucinous adenocarcinoma	18 (14)	22 (16)	21 (15)	16 (12)	
Differentiation	Well	28 (22)	23 (17)	35 (25)	31 (23)	0.202
	Moderate	75 (60)	81 (59)	87 (61)	89 (64)	
	Poor	22 (18)	33 (24)	20 (14)	18 (13)	
Lymph nodes involvement^*^		14.15±2.11	14.55±1.89	14.35±2.01	14.65±1.95	0.181
Surgical pathological TNM stage (related to AJCC staging system)	I	22 (18)	30 (21)	29 (20)	33 (23)	0.925
	II	64 (51)	53 (39)	61 (43)	55 (40)	
	III	38 (30)	53 (39)	51 (36)	49 (36)	
	IV	1 (1)	1 (1)	1 (1)	1 (1)	
QLQ-C30/CR38 score/patient		79.37±5.11	75.23±6.24	76.46±6.44	71.53±4.55	Clinically insignificant difference^**^

Categorical and ordinal variables are presented as frequency (percentages) and continuous variables are presented as mean ± standard deviation (SD)

Categorical and ordinal variables were analyzed by the Chi-square test for independence and continuous variables were analyzed by one-way ANOVA

A *p* value less than 0.05 was considered significant

*N/A* not applicable, *QLQ-C30/CR38* quality of life questionnaires-cancer specific 30/colorectal cancer specific 38, *TNM stage* Tumor, Node, and Metastasis stage, *AJCC staging system* American Joint Committee on Cancer staging system

^*^Mean number of retrieved lymph nodes/patient at pathological examination

^**^mean difference < 10 score

### Surgical checkpoints

**Fig. 2:**
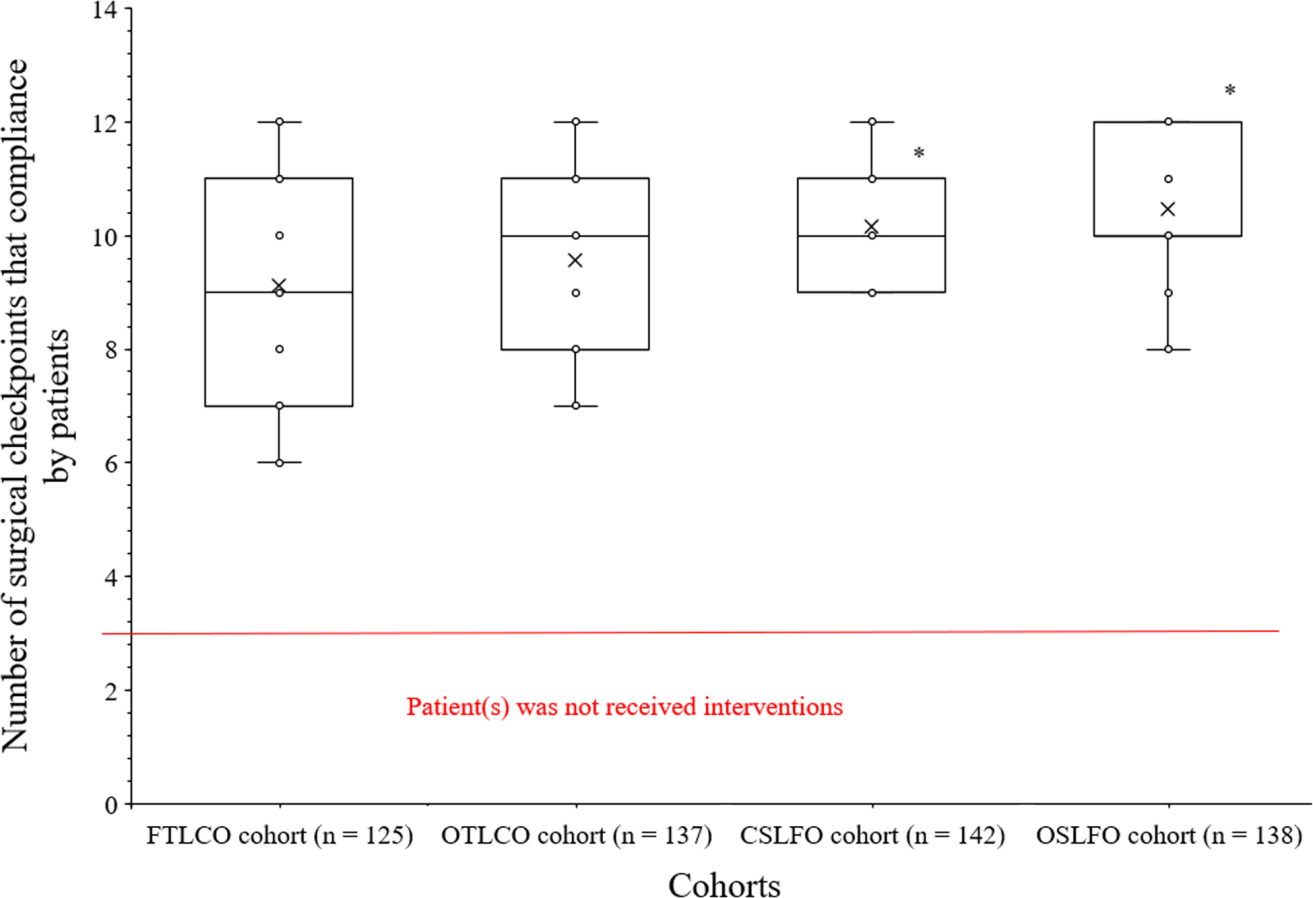
The number of surgical checkpoints that compliance by patient. Maximum of 13 checkpoints. *Higher number of surgical check-points compliance/patient than those of FTLCO and OTLCO cohorts

A high number of surgical checkpoints were compliance by patient of CSLFO cohort than those of FTLCO (10.17 ± 1.02/patient vs. 9.13 ± 1.86/patient, *p* < 0.0001, *q* = 8.218) and OTLCO (10.17 ± 1.02/patient vs. 9.56 ± 1.67/patient, *p* < 0.0001, *q* = 4.907) cohorts. A high number of surgical checkpoints were compliance by patient of OSLFO cohort than those of FTLCO (10.46 ± 1.18/patient vs. 9.13 ± 1.86/patient, *p* < 0.0001, *q* = 10.417) and OTLCO (10.46 ± 1.18/patient vs. 9.56 ± 1.67/patient, *p* < 0.0001, *q* = 7.181) cohorts. There was no statistically significant difference for surgical checkpoints compliance/patient between patients of the FTLCO cohort and those of the OTLCO cohort (*p* < 0.0001, *q* = 3.398). Also, there was no statistically significant difference for surgical checkpoints compliance/patient between patients of the CSLFO cohort and those of the OSLFO cohort (*p* < 0.0001, *q* = 2.329). The details of surgical checkpoints compliance by patients are presented in Fig. 2.

### Hospital stays

Patients of FTLCO cohort had shorter length of hospital stay than those of CSLFO (14.15 ± 2.06 days/patient vs. 30.47 ± 8.37 days/patient, *p* < 0.0001, *q* = 27.582) and OSLFO (14.15 ± 2.06 days/patient vs. 34.27 ± 10.12 days/patient, *p* < 0.0001, *q* = 33.769) cohorts. Patients of OTLCO cohort had shorter length of hospital stay than those of CSLFO (15.67 ± 2.12 days/patient vs. 30.47 ± 8.37 days/patient, *p* < 0.0001, *q* = 25.618) and OSLFO (15.67 ± 2.12 days/patient vs. 34.27 ± 10.12 days/patient, *p* < 0.0001, *q* = 31.962) cohorts. There was no statistically significant difference between lengths of hospital stay of patients of FTLCO cohort and those of OTLCO cohort (*p* < 0.0001, *q* = 2.253). Patients of CSLFO cohort had shorter length of hospital stay than those of OSLFO cohort (*p* < 0.0001, *q* = 6.583).

Patients of FTLCO cohort had shorter postoperative hospital stays than those of CSLFO (7.73 ± 1.38 days/patient vs. 10.96 ± 2.28 days/patient, *p* < 0.0001, *q* = 19.223) and OSLFO (7.73 ± 1.38 days/patient vs. 11.42 ± 2.42 days/patient, *p* < 0.0001, *q* = 21.829) cohorts. Patients of OTLCO cohort had shorter postoperative hospital stays than those of CSLFO (8.31 ± 1.35 days/patient vs. 10.96 ± 2.28 days/patient, *p* < 0.0001, *q* = 16.519) and OSLFO (8.31 ± 1.35 days/patient vs. 11.42 ± 2.42 days/patient, *p* < 0.0001, *q* = 18.846) cohorts. There was no statistically significant difference for postoperative hospital stays between patients of FTLCO cohort and those of OTLCO cohort (*p* < 0.0001, *q* = 3.145). Also, there was no statistically significant difference for postoperative hospital stays between patients of CSLFO cohort and those of OSLFO cohort (*p* < 0.0001, *q* = 2.825).

**Fig. 3:**
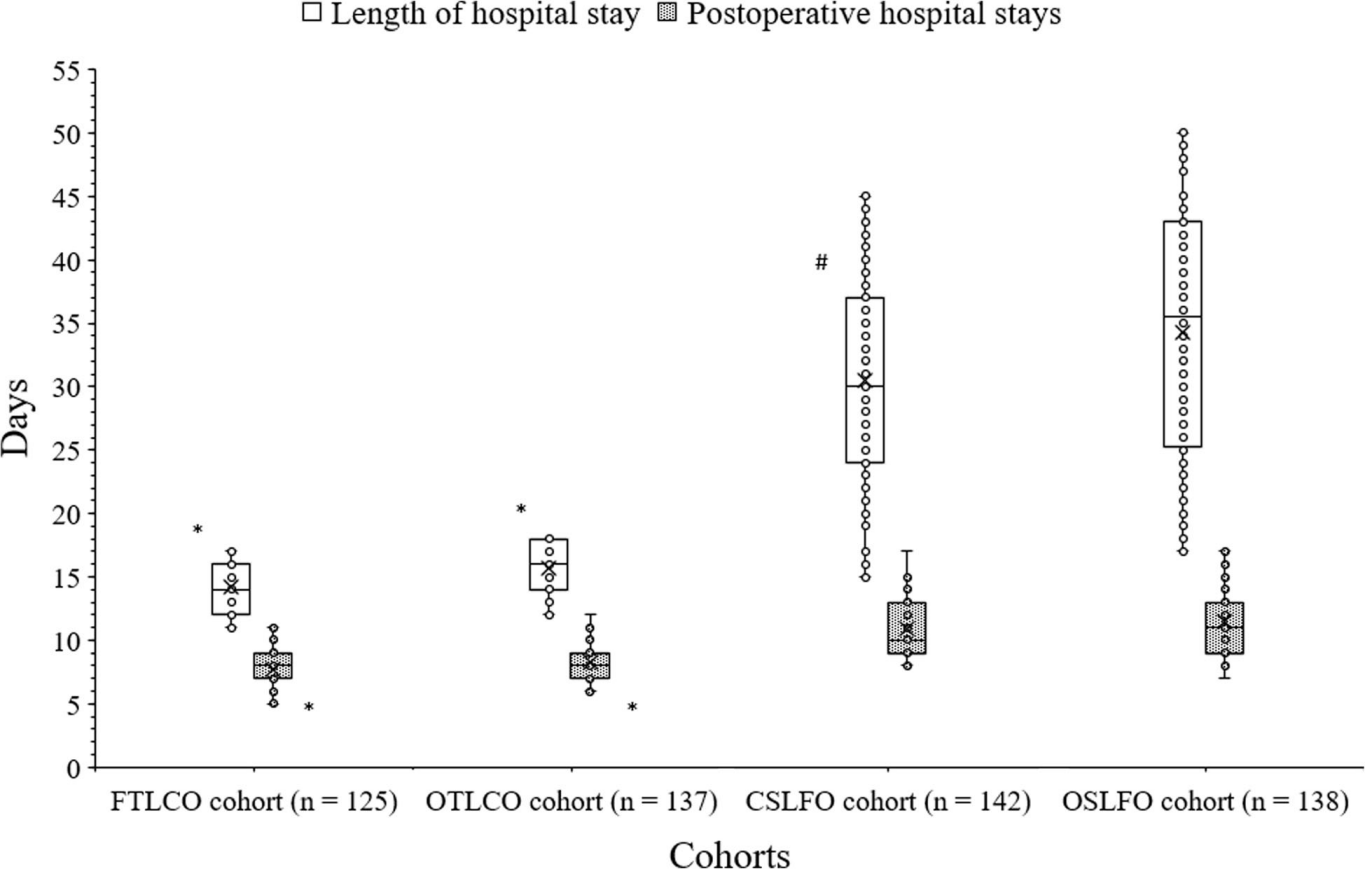
Hospital stays analysis. *Shorter days/patient than those of CSLFO and OSLFO cohorts. #Shorter days/patient than those of OSLFO cohort

The details of length of hospital stay and postoperative hospital stays are represented in Fig. 3.

### Outcomes for surgery

Flatus resumption time and the time until first defecation were shorter for the patients who underwent fast-track surgical procedures than those who underwent conventional surgical procedures (*p* < 0.05 for all). Those post-surgical outcomes were also shorter for patients who underwent the fast-track laparoscopy than those who underwent open fast-track surgical procedure (*p* < 0.05 for both). Resumption of a fluid diet time, intraoperative blood loss, and ambulation onset time were shorter for the patients who underwent fast-track surgical procedures than those who underwent conventional surgical procedures (*p* < 0.05 for all). Intraoperative blood loss was least for the patients who underwent fast-track laparoscopy surgeries than those who underwent other surgeries (*p* < 0.05). There were no significant differences for the number of the readmission of patients within 30 days among cohorts (*p* = 0.975). The detailed the outcomes for surgery are reported in Table 3.

**Table 3:** Outcomes for surgeries

Parameters	Cohorts				Comparisons						
	FTLCO	OTLCO	CSLFO	OSLFO							
Surgery	Fast-track laparoscopy	Open fast-track	Conventional laparoscopy	Open conventional	*p* value	*q* value					
Numbers of patients underwent surgeries	125	137	142	138		FTLCO vs. OTLCO	FTLCO vs. CSLFO	FTLCO vs. OSLFO	OTLCO vs. CSLFO	OTLCO vs. OSLFO	CSLFO vs. OSLFO
Flatus resumption time (hours/patient)	55.67±11.62	63.58±10.89	69.86±13.99	71.20±15.81	< 0.0001	6.818	12.329	13.407	5.85	3.764	1.198
The time until first defecation (days/patient)	76.15±12.21	81.15±13.22	91.15±15.87	93.87±16.88	< 0.0001	3.879	11.739	13.774	8.015	10.123	2.184
Resumption of a fluid diet time (days/patient)	28.09±11.05	30.15±13.41	55.41±13.41	58.61±15.42	< 0.0001	1.749	23.397	25.962	22.155	24.786	2.812
Ambulation onset time (hours/patient)	32.15±9.45	33.41±10.41	52.41±15.42	55.46±14.48	< 0.0001	1.126	18.259	20.867	17.537	20.209	2.819
Intraoperative blood loss (mL)	110±15	125±20	165±35	175±40	< 0.0001	5.777	21.361	25.075	15.909	19.748	3.985
The readmission of patients within 30 days	7 (6)	8 (6)	9 (6)	7 (5)	0.975	N/A	N/A	N/A	N/A	N/A	N/A

Categorical variables are presented as frequency (percentages) and continuous variables are presented as mean ± standard deviation (SD)

Variables were analyzed by one-way ANOVA

The Tukey test was used for post hoc analysis

A *p* value less than 0.05 and *q* value greater than 3.633 were considered significant

*N/A* not applicable

### Surgical cost

**Fig. 4:**
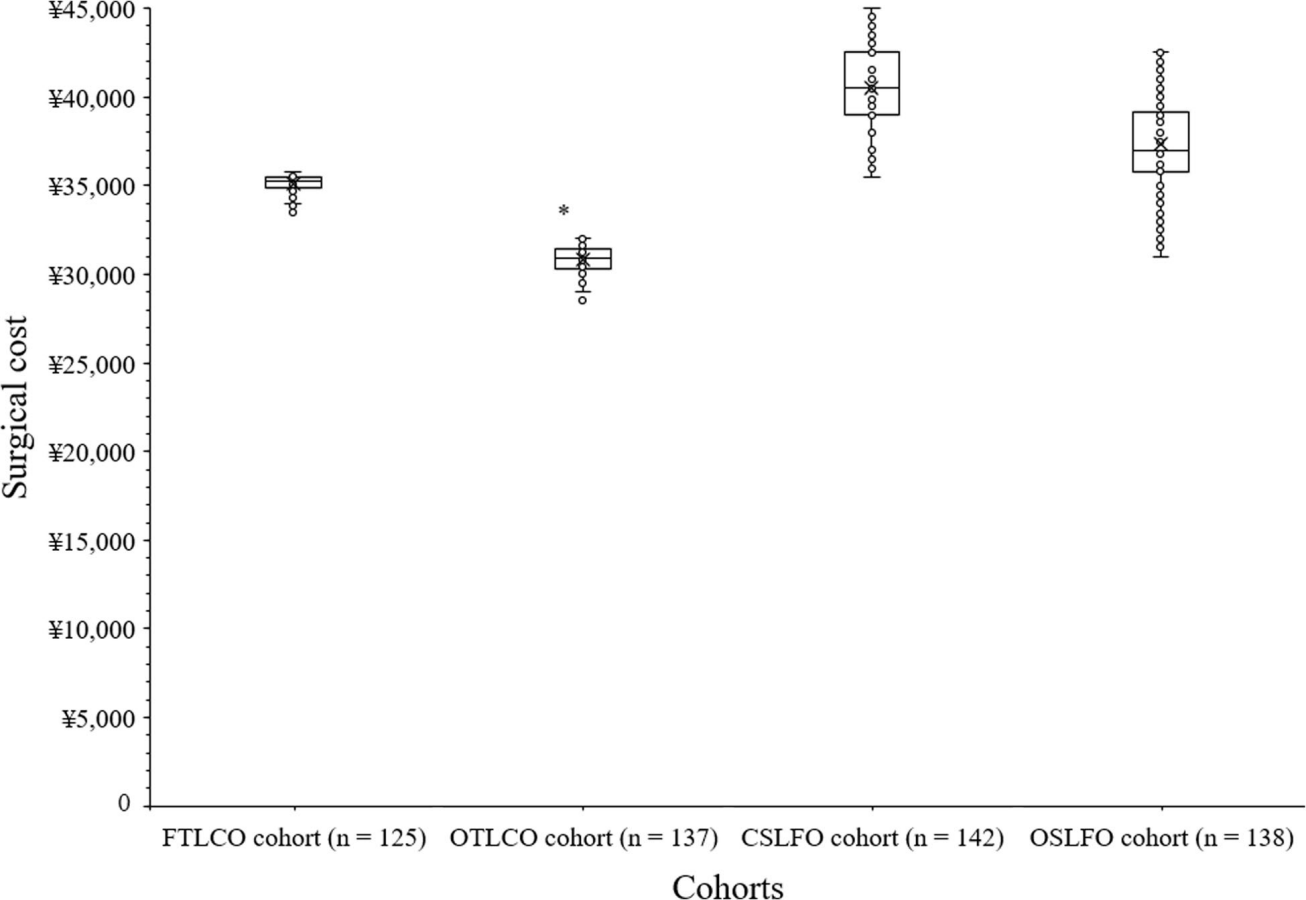
Surgical cost of patients. *Fewer ¥/patient than those of FTLCO, CSLFO, and OSLFO cohorts

The surgical cost was fewer for patients of OTLCO cohort than those of FTLCO (30,825 ± 731 ¥/patient vs. 35,125 ± 493 ¥/patient, *p* < 0.0001, *q* = 27.375), CSLFO (30,825 ± 731 ¥/patient vs. 40,475 ± 2,299 ¥/patient, *p* < 0.0001, *q* = 63.457), and OSLFO (30,825 ± 731 ¥/patient vs. 37,326 ± 2,544 ¥/patient, *p* < 0.0001, *q* = 42.446) cohorts. The surgical cost was fewer for patients of FTLCO than those of CSLFO (*p* < 0.0001, *q* = 34.353) and OSLFO (*p* < 0.0001, *q* = 14.037) cohorts. The surgical cost was fewer for patients of OSLFO cohort than those of CSLFO cohort (*p* < 0.0001, *q* = 20.748). The open fast-track surgeries were least expensive than the other surgeries. The details of surgical cost are presented in Fig. 4.

### Chemotherapy treatment

A total of 103, 107, 113, and 105 patients of FTLCO cohort, OTLCO cohort, CSLFO cohort, and OSLFO cohort received chemotherapy after post-operative discharge, respectively. There were no significant differences for the number of patients with any grade adverse effects (*p* = 0.431) and the number of patients with grade 3–4 grade adverse effects after chemotherapy (*p* = 0.858). The cost of chemotherapy was statistically the same for patients among cohorts (*p* = 0.151). The details of chemotherapy-emergent adverse effects and the cost of chemotherapy after post-operative discharge are reported in Table 4.

**Table 4:** Chemotherapy treatment

Parameters	Cohorts				Comparisons
	FTLCO	OTLCO	CSLFO	OSLFO	
Surgery	Fast-track laparoscopy	Open fast-track	Conventional laparoscopy	Open conventional	
Adjuvant chemotherapy	8 cycles of capecitabine and oxaliplatin; every 21 days	8 cycles of capecitabine and oxaliplatin; every 21 days	12 cycles of leucovorin, fluorouracil, and oxaliplatin; every 15 days	12 cycles of leucovorin, fluorouracil, and oxaliplatin; every 15 days	
Numbers of patients who received chemotherapy	103	107	113	105	*p* value
Any grade adverse effects	93 (90)	102 (95)	107 (95)	99 (94)	0.431
Grade 3–4 adverse effects	37 (36)	39 (36)	41 (36)	43 (41)	0.858
Cost (¥/patient)	99,000±10,000	98,500±17,500	102,000±15,000	101,500±10,500	0.151

Categorical and ordinal variables are presented as frequency (percentages) and continuous variables are presented as mean ± standard deviation (SD)

Variables were analyzed by one-way ANOVA

The Tukey test was used for post hoc analysis

A *p* value less than 0.05 and *q* value greater than 3.633 were considered significant

The National Cancer Institute Common Terminology Criteria for Adverse Events v4.03 was preferred for evaluation of the chemotherapy-related adverse events

*N/A* not applicable

### Quality of life

**Fig. 5:**
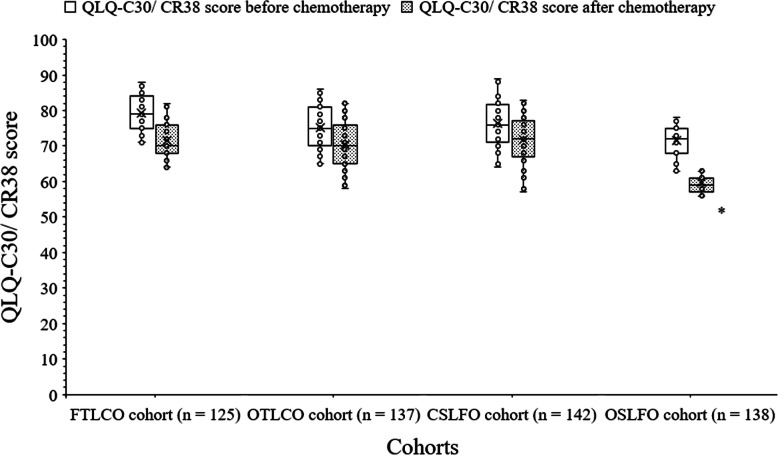
European Organization for Research and Treatment questionnaires score. The mean differences in QLQ-C30/CR38 (quality of life questionnaires-cancer specific 30/colorectal cancer specific 38) score of 10 or more points was considered as clinically significant difference. *Clinically significant lower QLQ-C30/CR38 score/patient than those of FTLCO cohort

QLQ-C30/CR38 score was clinically same (mean difference < 10 score) before surgery among all cohorts. It was clinically lower in case of patients of OSLFO cohort than those of FTLCO cohort after chemotherapy (59.63 ± 2.26 score/patient vs. 71.67 ± 5.19 score/patient, Fig. 5).

### Survival

A total follow-up period was 10–20 months (12 ± 3 months). Two patients from the OSLFO cohort died during follow-up. The disease-free survival and overall survival were the same for patients among cohorts.

## Discussion

The patients who underwent the fast-track surgical procedures had shorter length of hospital stay and postoperative hospital stays than those who underwent the conventional surgical procedures. The results of the lengths of hospital stay of the current study agreed with those of the LAFA-study [8] and the other available studies on the Chinese population [4, 10–14]. The fast-track surgical approach is first introduced in the medical oncology for multidiscipline treatments [14]. In the fast-track surgical approach the colorectal cancer is treated as an integrated disease but not as a surgical disease [4]. The fast-track surgical procedure is effective even in a multidisciplinary scenario as colorectal cancer treatment in which surgery is only a part of management.

The laparoscopic surgical procedures did not reduce the length of hospital stay and postoperative hospital stays than open surgical procedures. Also, the disease-free survival and overall survival were the same between the fast-track surgical procedures and the conventional surgical procedures. The results of the length of hospital stay and survival of the current study were consistent with those of the trial [4]. The fast-track surgical procedures are feasible and have advantages over open surgical procedures for right and left colon cancer [18]. The fast-track surgical approach is as safe as the conventional surgical procedures.

The laparoscopic surgical procedures reduced the flatus resumption time, the time until first defecation, and the intraoperative blood loss. The results of the surgical outcome measures of the current study agreed with those of the other available studies [4, 17]. During laparoscopies, surgeons efficiently dissect tumors and have high-definition views [4]. The laparoscopic surgical procedures have favorable effects for patients with colorectal cancer than open surgical procedures.

The study is reported that the open fast-track surgeries were least expensive than the other surgeries. The results of the cost of the surgeries of the current study agreed with those of the LAFA-study [8] and the other available study [4]. Thus, the open fast-track surgeries are better choice for colorectal cancer treatment than the conventional laparoscopic surgical procedures or the open conventional surgical procedures.

The study reported that there were no significant differences for the number of patients with any grade adverse effects or with grade 3–4 adverse effects and QLQ-C30/CR38 score among cohorts. Adjuvant chemotherapy would not affect the quality of the post-operative recovery irrespective of which pathway offered to the patients. The tolerability of chemotherapeutic agent(s) varies greatly from one case to another regardless the kind of surgery or recovery pathway offered to the patients 3–4 weeks before starting chemotherapy. However, patients of FTLCO cohort had clinically better QLQ-C30/CR38 score (changes in 10 or more score) than those of OSLFO cohort after chemotherapy. The justification for the same is that both groups of patients were treated with different chemotherapy regimens.

In the limitation of the study, for example, retrospective study and lack of randomized trial. The surgical checkpoints that were compliance by the patient of fast-track surgeries were significantly fewer than those of the conventional surgeries. The postoperative hospital stays of patients in the fast-track groups and those of the standard care groups seems to be long for patients with an uncomplicated course. Also, CSLFO and OSLFO patients had statistically similar postoperative hospital stays. The study did not include the chemotherapy-related hospital stay in the assessment. The possible justification for the same is the chemotherapy-related hospital stay was fixed (3 days for the conventional surgical procedures and 1 day for the fast-track surgical procedures) regardless the kind of the conditions of patients. No information about how patients might have been allocated to the open or laparoscopic procedures and to have or not have fast-track recovery protocols initiated, and how this might have biased the results is not discussed. Chemotherapy dose used as adjuvant treatment in the current study was less than the usually accepted regimens. The disease-free survival and overall survival were the same between the fast-track surgical procedure and the conventional procedure. These conclusions are not entirely correct. A logistic regression could be performed for survival since the groups received different chemotherapy and different surgery but the study lacks a logistic regression analysis.

## Conclusions

The open fast-track surgeries are less expensive. The laparoscopic surgical procedures have favorable effects for patients with colorectal cancer than the open surgical procedures. The fast-track surgical approach is as safe as the conventional surgical procedures and has more favorable effects than the conventional surgical procedures. The study is recommending laparoscopic fast-track surgical procedures for colorectal cancer treatment. The study reported that there are advantages when adopting early recovery protocols after colorectal cancer surgery, even in patients referred for neoadjuvant chemotherapy treatment. The finding will help the oncologists to provide information about the diffusion of enhanced recovery pathways in China. The study includes high quality and scientifically sound works exploring new surgical technologies and innovative surgical techniques that have the possibility to improve patient care and push the boundaries of surgery.

## Data Availability

The datasets used and analyzed during in this study are available from the corresponding author on reasonable request.

## Abbreviations

ANOVA: Analysis of variance
*q*: The critical value for Tukey test
QLQ-C30/CR38: Quality of life questionnaires-cancer specific 30/colorectal cancer specific 38
SD: Standard deviation
TNM stage: Tumor, Node, and Metastasis stage
AJCC staging system: American Joint Committee on Cancer staging system
